# Intrauterine Pregnancy With a Correctly Positioned Levonorgestrel-Releasing Intrauterine System: A Case Report

**DOI:** 10.7759/cureus.110479

**Published:** 2026-06-08

**Authors:** Jaroslaw Kopko, Polina Novikava, Kamil Sobocinski

**Affiliations:** 1 Department of Obstetrics and Gynecology, Lazarski University of Warsaw, Warsaw, POL; 2 Department of Obstetrics and Gynecology, Medical University of Białystok, Bialystok, POL; 3 Department of Gynecology and Oncological Gynecology, Military Institute of Medicine, Warsaw, POL

**Keywords:** contraceptive failure, hysteroscopy, intrauterine pregnancy, levonorgestrel intrauterine system, lng-ius, miscarriage

## Abstract

Intrauterine pregnancy occurring with a correctly positioned levonorgestrel-releasing intrauterine system (LNG-IUS) is extremely rare. Most pregnancies associated with LNG-IUS use result from device malposition, unnoticed expulsion, or insertion during an undiagnosed early pregnancy. Pregnancy with an intrauterine device in situ is associated with an increased risk of miscarriage, preterm delivery, and intra-amniotic infection.

We report the case of a 39-year-old gravida 4 para 2 abortus 1 woman with a correctly positioned 52-mg LNG-IUS who presented at 12 weeks’ gestation with leakage of amniotic fluid. One week earlier, an ultrasound had confirmed a viable intrauterine pregnancy with correct device positioning in the uterine fundus. Because the IUS strings were not visible on examination, no immediate removal was attempted. The patient wished to continue the pregnancy. During hospitalization, intrauterine fetal demise occurred, followed by pharmacological induction of miscarriage and uterine curettage with device removal.

This case highlights the clinical challenges of pregnancy with an LNG-IUS in situ and emphasizes that the lack of visible strings should not preclude consideration of removal. Current evidence suggests that hysteroscopic removal in early pregnancy may reduce adverse outcomes and should be considered in patients wishing to continue pregnancy.

## Introduction

Intrauterine devices (IUDs) are among the most effective reversible contraceptive methods. The two most commonly used types are copper IUDs and levonorgestrel-releasing intrauterine systems (LNG-IUS) [1]. The reported Pearl Index is approximately 0.16 for the 52-mg LNG-IUS [2]. Most pregnancies associated with LNG-IUS use result from device malposition, unnoticed expulsion, or insertion during an undiagnosed early pregnancy [3,4]. Intrauterine pregnancy with a correctly positioned LNG-IUS is extremely rare. Approximately 53% of pregnancies occurring with LNG-IUS are ectopic [5]. Pregnancy with an IUD in situ is associated with increased risks of miscarriage, preterm delivery, and intra-amniotic infection [6-8]. Evidence suggests that IUD removal, either by forceps or hysteroscopy, may improve outcomes, particularly when strings are not visible [9,10].

## Case presentation

**Figure 1:**
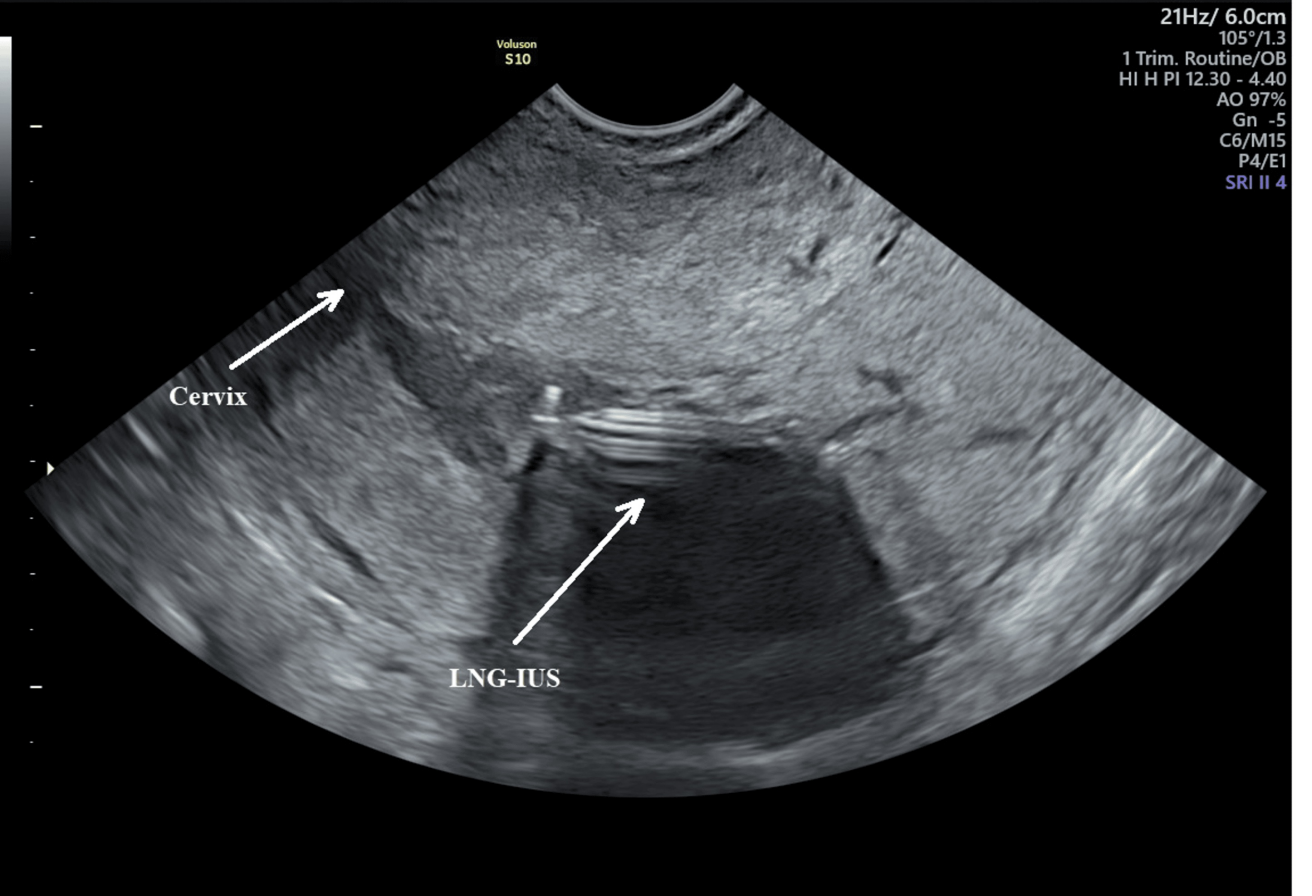
Ultrasound visualization of the correctly positioned levonorgestrel-releasing intrauterine system (LNG-IUS) adjacent to the gestational sac.

**Figure 2:**
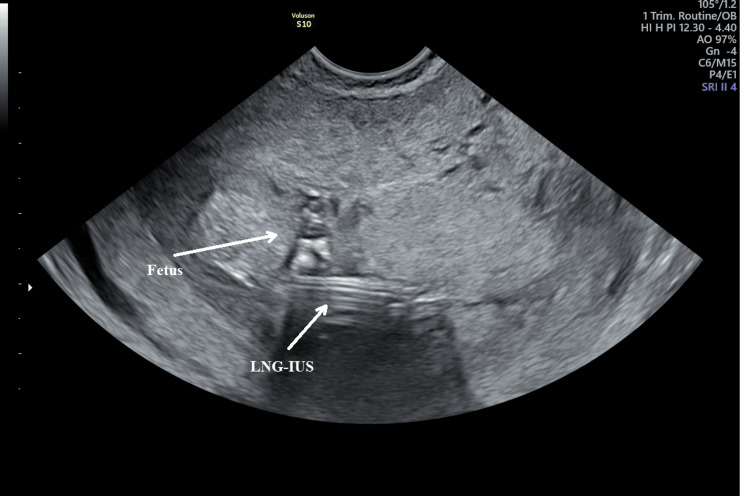
Ultrasound image demonstrating a viable intrauterine pregnancy at 12 weeks’ gestation with the LNG-IUS located in the uterine fundus. LNG-IUS: levonorgestrel-releasing intrauterine system.

A 39-year-old gravida 4 para 2 abortus 1 woman with a correctly positioned 52-mg LNG-IUS was admitted at 12 weeks’ gestation due to leakage of amniotic fluid. Ultrasound confirmed a single viable intrauterine pregnancy with a fetal heart rate of 160 beats/min and crown-rump length of 64 mm, consistent with 12 weeks and three days of gestation. The LNG-IUS was visualized in the uterine fundus (Figures 1 and 2).

Speculum examination showed no visible device strings. The patient had no relevant medical history, had previously delivered two children vaginally, and had one spontaneous miscarriage. The LNG-IUS had been inserted 10 months earlier, with follow-up confirming correct placement. Menstrual bleeding persisted after insertion but became lighter and shorter. In the weeks preceding admission, the patient reported persistent amenorrhea and symptoms suggestive of pregnancy despite the presence of an LNG-IUS. For this reason, gynecological evaluation and ultrasound examination were performed. One week before admission, an ultrasound unexpectedly confirmed a viable intrauterine pregnancy with normal amniotic fluid volume and a correctly positioned LNG-IUS. Because the strings were not visible on speculum examination, simple office removal was not feasible. Conservative management was initially chosen after counseling regarding the potential risks of intervention and the uncertain benefits and risks associated with invasive removal procedures during an ongoing pregnancy. On admission, the patient was counseled regarding the poor prognosis but wished to continue the pregnancy. She received antibiotics, hydration, and analgesia due to increasing inflammatory markers. Intrauterine fetal demise occurred during hospitalization. Medical induction of miscarriage followed by uterine curettage was performed, and the LNG-IUS was removed. The patient was discharged in good condition.

## Discussion

The LNG-IUS is a highly effective long-acting reversible contraceptive with a reported first-year failure rate of approximately 0.1% [11]. Large cohort data confirm its very low pregnancy incidence [5]. Furthermore, among pregnancies occurring during LNG-IUS use, approximately half are reported to be ectopic, highlighting the rarity of the intrauterine pregnancy observed in our patient. Several risk factors for contraceptive failure have been identified in the literature, including device malposition, unnoticed expulsion, insertion during an unrecognized early pregnancy, and failure to confirm correct device placement after insertion [12-15]. In contrast, none of these factors was identified in the present case. Correct positioning was documented both after insertion and during pregnancy, highlighting the exceptional rarity of this presentation. Pregnancy with an IUD in situ significantly increases risks of miscarriage, preterm delivery, and intra-amniotic infection [6-8]. A systematic review by Brahmi et al. demonstrated that removal of an IUD during early pregnancy substantially reduces the risk of spontaneous miscarriage and adverse pregnancy outcomes compared with retaining the device [6]. Although the risks do not return to baseline after removal, outcomes are consistently better than when the IUD remains in situ. Early removal, therefore, appears to improve pregnancy outcomes [9]. When strings are not visible, hysteroscopic removal may be considered. In the present case, conservative management was initially chosen because the device strings were not visible on examination, precluding simple office removal. At the time of diagnosis, the patient had already reached the late first trimester, and the potential benefits of hysteroscopic removal had to be weighed against the risk of procedure-related pregnancy loss. However, current evidence suggests that referral for hysteroscopic removal in specialized centers may still be appropriate in selected patients wishing to continue pregnancy. A 2022 scoping review reported that hysteroscopic removal in early pregnancy is feasible and relatively safe in experienced hands, with approximately 10% risk of miscarriage and 12% risk of preterm delivery associated with the procedure [16]. This case underscores that the absence of visible strings should not automatically preclude consideration of hysteroscopic removal in specialized centers. This case reinforces the importance of maintaining clinical suspicion for pregnancy in women using highly effective contraception. Ultrasound evaluation should be considered in women presenting with amenorrhea, abnormal bleeding, pelvic pain, pregnancy-related symptoms, or uncertainty regarding device position.

## Conclusions

Pregnancy with a correctly positioned LNG-IUS is extremely rare. It is associated with significant maternal and fetal risks. When strings are not visible and the patient wishes to continue pregnancy, hysteroscopic removal should be considered, as current evidence suggests that it may improve outcomes.
